# Infection Patterns of a Liberibacter Associated with Macrohomotoma gladiata, a Psyllid Feeding on Ficus microcarpa

**DOI:** 10.1128/spectrum.03614-22

**Published:** 2022-12-01

**Authors:** Fang-Yu Lin, Shin Lee, Yi-Chang Liao, Man-Miao Yang, Chia-Ching Chu

**Affiliations:** a Department of Plant Pathology, National Chung Hsing University, Taichung, Taiwan; b Department of Entomology, University of California, Riverside, California, USA; c Department of Entomology, National Chung Hsing University, Taichung, Taiwan; d Advanced Plant Biotechnology Center, National Chung Hsing University, Taichung, Taiwan; USDA–San Joaquin Valley Agricultural Sciences Center

**Keywords:** *Candidatus* Liberibacter, distribution pattern, infection density, *Macrohomotoma gladiata*, psyllid

## Abstract

Almost all known Liberibacters can be transmitted by psyllids. This suggests that there is a coevolutionary relationship between these two groups of organisms. However, detailed investigation of Liberibacters and psyllids have often focused on only a few species, thus potentially limiting knowledge on Liberibacter-psyllid associations. This study investigated the infection patterns of a Liberibacter inhabiting Macrohomotoma gladiata, a psyllid species feeding on Ficus microcarpa. Comparison of the Liberibacter’s near-full-length 16S rDNA sequence with those of other known Liberibacters revealed that it is closely related to *Candidatus* Liberibacter asiaticus. A survey of different *M*. *gladiata* populations in Taiwan using conventional and quantitative PCR (qPCR) indicated that the Liberibacter could be detected with variable frequencies in all the tested populations; the proportions of individuals carrying large Liberibacter populations also differed depending on the population. Additional analysis of a larger set of samples collected from one specific population revealed that the psyllid’s gender and abdominal color were associated with Liberibacter infection density. Significantly greater proportions of individuals with a blue/green abdomen carried high Liberibacter titers. Analysis of the psyllids’ body lengths revealed that body size was not affected by Liberibacter infection status and that females, particularly those with an orange abdomen, tended to be larger. The infection patterns of Liberibacter in nymph-infested and nymph-free twigs of *F*. *microcarpa* were also determined, and Liberibacter distribution was found to be associated with the presence of nymphs. These findings broaden the understanding of Liberibacter ecology in general and have implications for managing Liberibacter-associated diseases.

**IMPORTANCE** Despite the ever-increasing interest in Liberibacter-psyllid interactions, most of the current knowledge on the subject has been established from studies focusing on species associated with crop diseases. To obtain a more holistic understanding of Liberibacter ecology, we investigated the infection patterns of a Liberibacter recently detected in *Macrohomotoma gladiata*, a psyllid pest of *Ficus microcarpa*. We showed that a Liberibacter closely related to *Candidatus* Liberibacter asiaticus is widely distributed across *M*. *gladiata* populations in Taiwan. The study also identified factors associated with the Liberibacter infection patterns, both in *M*. *gladiata* and in *F*. *microcarpa*. The effects of Liberibacter infection status on psyllid body sizes were also examined. Some of the patterns detected in this work were similar those found in well-known Liberibacters, while some were the opposite. The findings in this work broaden our understanding of Liberibacter ecology in general and may facilitate development of strategies for managing plant diseases.

## INTRODUCTION

Species of the genus *Candidatus* Liberibacter are a group of plant-associated bacteria; these bacteria mainly reside in the plant phloem and in some cases induce systemic symptoms on the plant hosts (1–3). For example, *Ca*. Liberibacter asiaticus is known as a putative causal agent of the citrus greening disease (4). Affected plants can exhibit symptoms ranging from leaf chlorosis, lopsided fruits, and weakening and even death of the entire tree, resulting in significant yield loss across different citrus-producing areas (4, 5). Other than Liberibacters that are putative plant pathogens, some do not seem to be detrimental to their hosts; *Ca.* Liberibacter europaeus and *Ca.* Liberibacter brunswickensis, which have been detected in psyllids inhabiting pear and *Solanum* spp., respectively, have not been associated with plant symptoms (6, 7). It is possible that these Liberibacters could behave like endophytes in these plants (6). The species *Ca.* Liberibacter europaeus has also been detected in Scotch broom and psyllids feeding on the plant (8, 9); in these hosts, however, symptoms such as stunting, leaf dwarfing, and leaf chlorosis were observed, suggesting that the same Liberibacter may have distinct effects on different plants.

Almost all known Liberibacters can be transmitted by insects belonging to the superfamily Psylloidea (i.e., psyllids or jumping plant lice) (5, 6, 10). These insect vectors are sap-sucking hemipterans that can live on a great variety of host plants (11). In general, psyllids exhibit strong host specificity (12). Reports on psyllid-Liberibacter associations have found that the nymphs and adults can acquire Liberibacter while feeding on the phloem sap and transmit these bacteria in a circulative, persistent, and propagative manner (13). Several studies have also documented fitness or behavioral changes resulting from infection by some Liberibacters For example, *Ca*. Liberibacter asiaticus-infected Asian citrus psyllids (Diaphorina citri) exhibit greater fertility and fitness than uninfected ones (14). However, infected adults have a shorter life span, and the rate of nymph development is reduced (14). Other studies have also shown that *Ca*. Liberibacter asiaticus can manipulate metabolic pathways, flight capacity, and even mate selection in *D. citri*. (15, 16). In the case of the potato psyllid (Bactericera cockerelli), infection by *Ca.* Liberibacter solanacearum reduces its fecundity but does not pose a detectable effect on adult life span (17). Also, potato psyllids exhibit more active probing behavior when they are infected by *Ca.* Liberibacter solanacearum (18).

Given these findings and the fact that all known insect vectors of Liberibacter to date belong to Psylloidea, it is likely that there is coevolutionary adaptation between these two groups of organisms (4, 19); this makes them interesting subjects for evolutionary and ecological studies. Despite significant interest in psyllid-Liberibacter interactions, detailed information on Liberibacters and their psyllid vectors is mostly limited to a few species and pathosystems, and therefore exploration of other similar interactions could help expand the understanding of these subjects.

**FIG 1:**
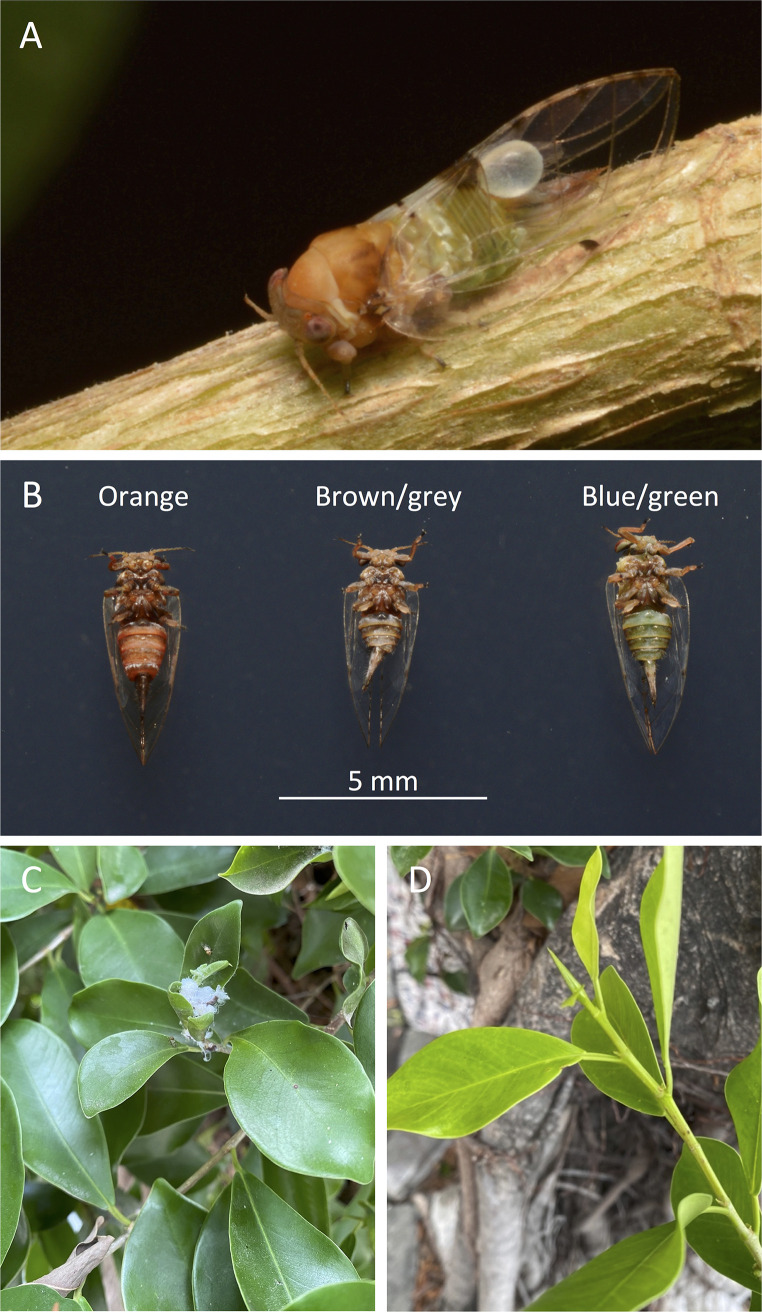
Insects (*Macrohomotoma gladiata*) and plant tissues (*Ficus microcarpa*) collected in this study. (A) An adult *M*. *gladiata*. (B) Adults with different abdominal colors. (C) A twig infested by nymphs. (D) An uninfested twig.

Macrohomotoma gladiata is a psyllid originally belonging to the family Homotomidae (20). Recently, the species has been reclassified to the family Carsidaridae (21). The species has five nymphal stages and produces more than one generation per year (22). They are native to areas including Taiwan, Japan, and Hong Kong (23, 24). The distribution of this species has also expanded to other parts of the world, including Spain, Italy, the United States, and northern Africa (25–29). In Taiwan, adult *M*. *gladiata* can be found exhibiting different abdominal colors, ranging from orange to brown/grey to blue/green (Fig. 1B). The plant host of *M*. *gladiata* is Ficus microcarpa (24), and so far, *M*. *gladiata* has been found only on this plant species (25–30). The nymphs and adults of *M*. *gladiata* exhibit different preferences for feeding sites (22). Nymphs are typically distributed on the inner and outer shoots, while adults commonly feed on the underside of outer leaves or the shoots (22). During oviposition, females lay eggs below the stipules of young leaves (22). Although *M*. *gladiata* is usually not considered a major pest, intense feeding can sometimes lead to death of the shoots (27). The nymphs can produce white, waxy secretions around the apical shoots, which often leads to the growth of sooty molds (22, 27).

In a preliminary survey of the presence of Liberibacter in field psyllids in Taiwan (in 2019) using genus-specific primers developed in a previous work (7), Liberibacter was detected in *M*. *gladiata* sampled in Taiwan. A recently published study also detected the presence of Liberibacter using Illumina amplicon sequencing; based on the obtained 402-bp 16S rDNA fragment, it was found that among the currently known Liberibacters, the bacterium detected in *M*. *gladiata* was most closely related to *Ca*. Liberibacter asiaticus (31).

Given the increasing interest in Liberibacter (3) and the fact that *M*. *gladiata* has become a pest in different parts of the world (25–29, 32), more research on the ecology of Liberibacter in *M*. *gladiata* is needed. The objective of this work is to investigate the infection patterns of Liberibacters in *M*. *gladiata* populations in Taiwan. Conventional and quantitative PCR (qPCR) assays targeting the bacterium were conducted to determine Liberibacter’s prevalence and infection densities in *M*. *gladiata*. Factors potentially associated with Liberibacter’s infection patterns were also identified. Finally, this work also tested whether Liberibacter’s distribution in *F. microcarpa* is associated with the nymphs’ distribution on the shoots. Overall, the findings from this study could broaden the understanding of field infection patterns of Liberibacter and provide suggestions for disease management.

## RESULTS

### A Liberibacter was detected in all *M*. *gladiata* populations tested.

To determine the presence and infection patterns of Liberibacter in different *M*. *gladiata* populations, adult psyllids were collected from five locations in 2019 and 2021 (Table 1) and tested using the Liberibacter-specific primers LG774F/LG1463R (7). A Liberibacter-specific amplicon was detected in all five *M*. *gladiata* populations, with variable frequencies. The proportions of Liberibacter-positive individuals (including both genders) detected in populations TC1, TC2, and TC3 were 77%, 67%, and 29% respectively; those in populations TP and TN were 58% and 84%, respectively. Sequences obtained from 40 Liberibacter-positive samples were identical (611/611 bp; GenBank accession number OP159066); the numbers of samples sequenced for TC1, TC2, TC3, TP, and TN were 17, 8, 5, 5, and 5, respectively. BLASTn results showed that the detected sequence shared high sequence similarity with known Liberibacters, such as *Ca*. Liberibacter asiaticus (see Table S1 in the supplemental material).

**TABLE 1:** Details of the psyllid populations collected in this work

Yr of sampling	Location (abbreviation of population name, if applicable)	Coordinates	Sample size (*n*)
2019	West District, Taichung (TC1)	24°08′11.0″N 120°40′31.0″E	22 (12 males and 10 females)
2019	South District, Taichung (TC2)	24°07′43.8″N 120°40′14.8″E	15 (12 males and 3 females)
2019	South District, Taichung (TC3)	24°07′25.7″N 120°40′32.4″E	21 (14 males and 7 females)
2021	Daan District, Taipei (TP)	25°01′49.7″N 121°32′09.8″E	12 (6 males and 6 females)
2021	North District, Tainan (TN)	23°00′15.1″N 120°12′42.1″E	25 (14 males and 11 females)

### The Liberibacter detected in *M*. *gladiata* is most closely related to *Ca*. Liberibacter asiaticus.

**FIG 2:**
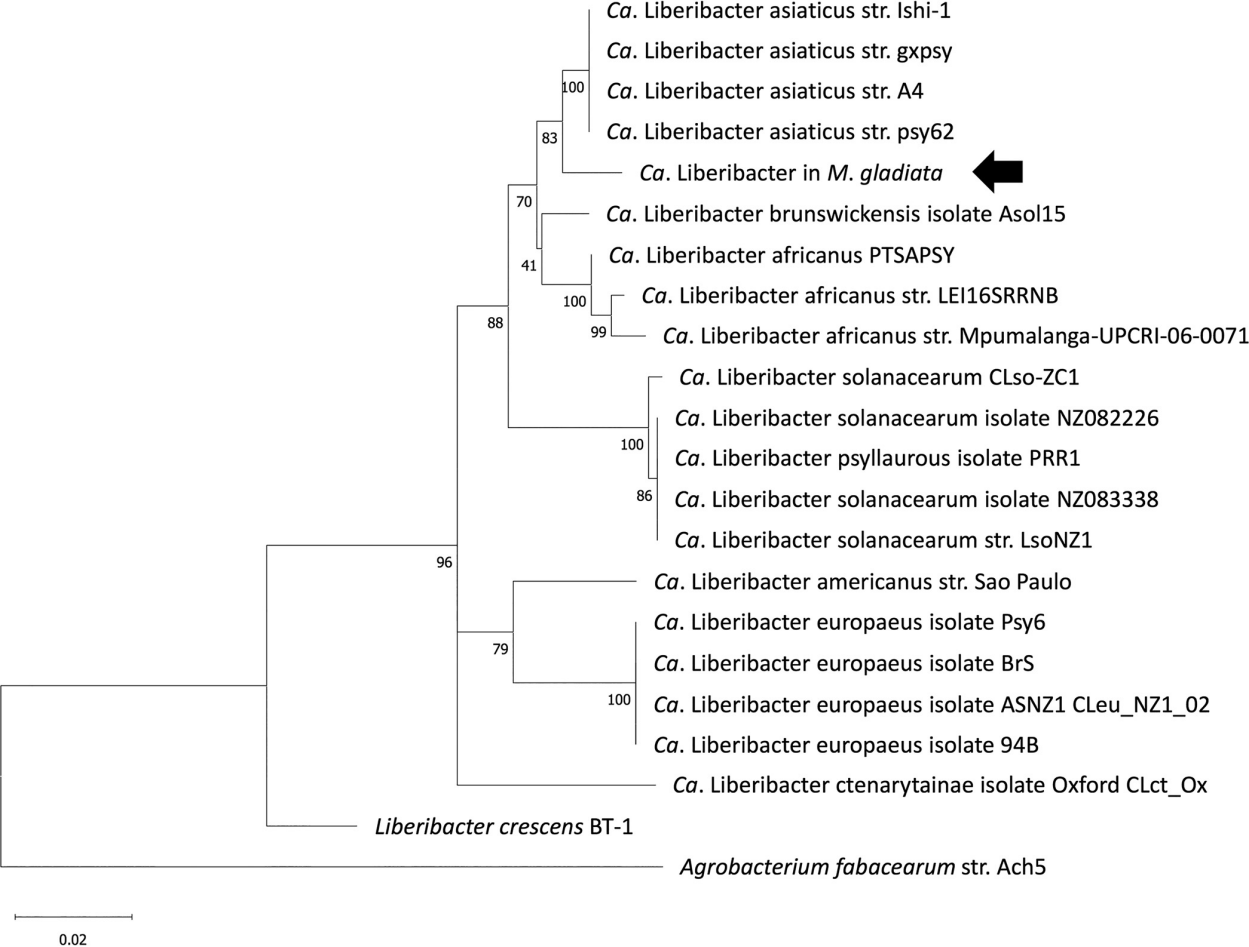
Phylogenetic tree reconstructed from near-full-length 16S rDNA sequences of different Liberibacters. The sequences of the Liberibacter discovered in *Macrohomotoma gladiata* (indicated with an arrow) and those of other known Liberibacters were subjected to a maximum-likelihood analysis. Agrobacterium fabacearum strain Ach5 was included as an outgroup.

To determine the phylogenetic placement of the Liberibacter detected in *M*. *gladiata*, two female psyllid DNA samples collected from the TC1 population (sampled in 2019) that produced large amounts of amplicons in the PCR assay described above were subjected to clone library construction using universal bacterial 16S rDNA primers 27f and 1492r (33). Near-full-length 16S rDNA sequences of the Liberibacter were obtained, and a maximum-likelihood analysis conducted in this work showed that among the Liberibacters included in the analysis (Table S2), the Liberibacter detected in *M. gladiata* was most closely related with *Ca*. Liberibacter asiaticus (Fig. 2), although the sequence identity between the detected Liberibacter and *Ca*. Liberibacter asiaticus was only 98.31%. The 16S rDNA sequence (1,421 bp; GenBank accession no. OP159054) was also confirmed to include the 611-bp sequence obtained from the Liberibacter-specific PCR tests described above.

### Proportions of *M. gladiata* adults with different Liberibacter infection statuses varied depending on the population tested.

**FIG 3:**
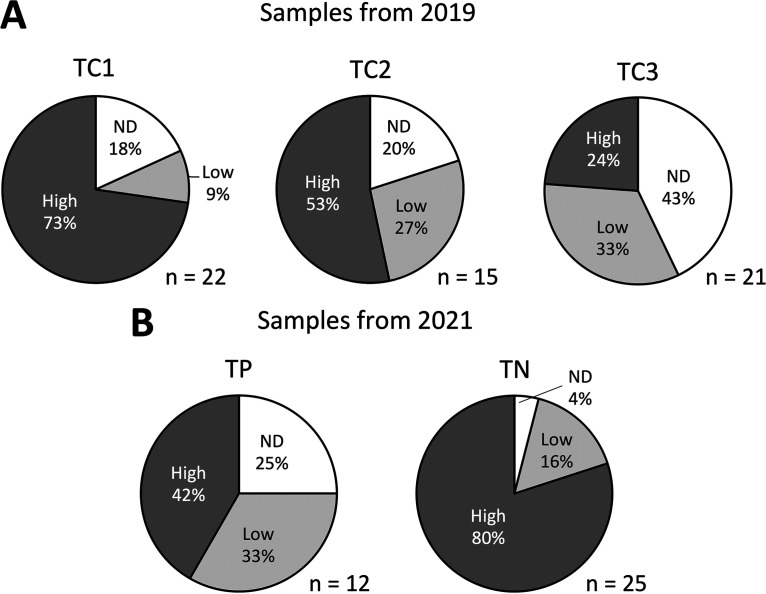
Proportions of *Macrohomotoma gladiata* individuals carrying different levels of Liberibacter load in different populations. (A and B) Psyllids obtained from (A) Taichung (TC1 to TC3) were sampled in 2019, and those obtained from (B) Taipei (TP) and Tainan (TN) were sampled in 2021. Liberibacter infection density was calculated by dividing each sample’s Liberibacter 16S rDNA copy number by that of *M. gladiata*’s glycerol kinase gene. n, number of *M. gladiata* adults; ND, not detected; low, infection density lower than 0.1; high, infection density higher than 0.1.

To determine the Liberibacter infection patterns across populations, qPCR assays targeting Liberibacter and *M*. *gladiata* genes were conducted. For each sample, the Liberibacter gene’s copy number was divided by that of the psyllid’s glycerol kinase gene. The resulting infection density values were then grouped into three categories: (i) undetected (ND), (ii) low-density infection (below 0.1), and (iii) high-density infection (above 0.1). In samples collected in 2019, a Fisher-Freeman-Halton test revealed that there were significant differences among the Liberibacter infection statuses of the three populations collected from Taichung (*P = *0.024; Fig. 3A). Among the three populations, TC1 had the highest proportion of psyllids infected with high densities of Liberibacter; the lowest proportion was found in population TC3. For samples obtained in 2021, the Liberibacter infection statuses between samples collected from Taipei (TP) and Tainan (TN) were also significantly different (*P = *0.032; Fisher-Freeman-Halton test; Fig. 3B). Samples collected from Tainan had higher proportions of psyllids with high-density infection.

As expected, the proportions of Liberibacter-positive samples in the more sensitive qPCR assays (low- and high-density infections combined) were higher than those in the conventional PCR tests using primer set LG774F/LG1463R.

### Abdominal color is associated with Liberibacter infection density in *M. gladiata*.

**FIG 4:**
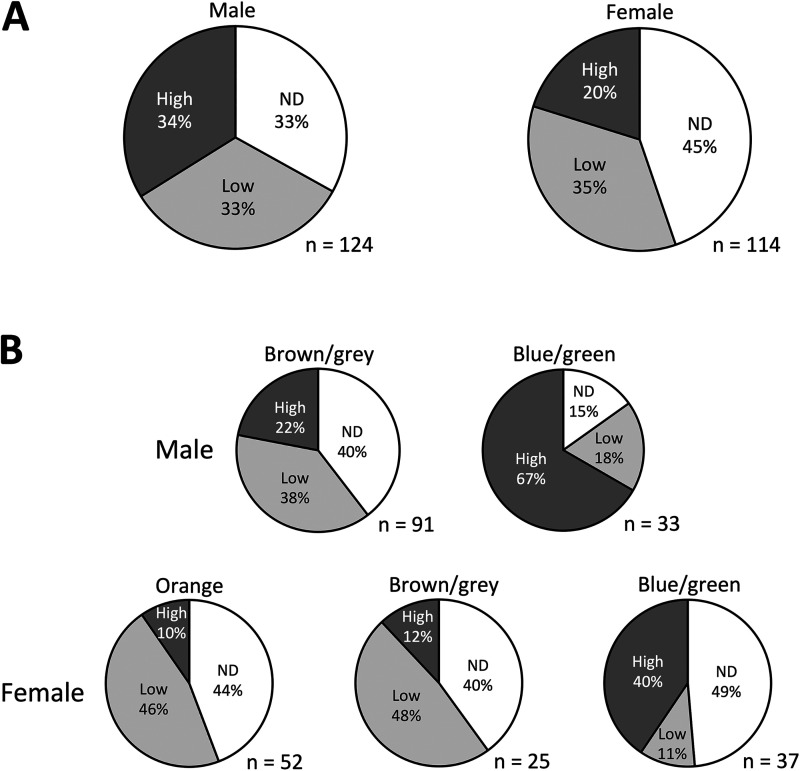
Liberibacter infection densities in *Macrohomotoma gladiata* separated by different factors. (A) Proportions of male and female *M. gladiata* carrying different Liberibacter loads. (B) Examination of the associations among gender, abdominal color, and Liberibacter infection density in *M. gladiata*. n, number of *M. gladiata* adults; ND, not detected; low, infection density lower than 0.1; high, infection density higher than 0.1.

To identify factors associated with Liberibacter infection status in *M*. *gladiata*, a larger number of *M. gladiata* adults (*n* = 238; 124 males and 114 females) were sampled from the TC2 population in 2020 and subjected to qPCR testing and data analysis. A significant difference in the Liberibacter infection status was detected between males and females of *M*. *gladiata* (*P = *0.044; Chi-square test; Fig. 4A). Compared to females, males had larger proportions of individuals carrying high titers of Liberibacter, although the deviation was not significant in the adjusted residual analysis (*P = *0.107 after the more conservative Bonferroni correction). In both genders, psyllids with a different abdominal color also shared a significant difference in Liberibacter infection densities (Chi-square test: male, *P < *0.001; female; *P < *0.001; Fig. 4B). Psyllids with a blue/green abdomen had significantly larger proportions of individuals with high-density infection compared to the other morphotypes (male, *P < *0.001; female, *P = *0.0015; Bonferroni-adjusted).

### Body length of *M. gladiata* is not associated with Liberibacter infection status.

**FIG 5:**
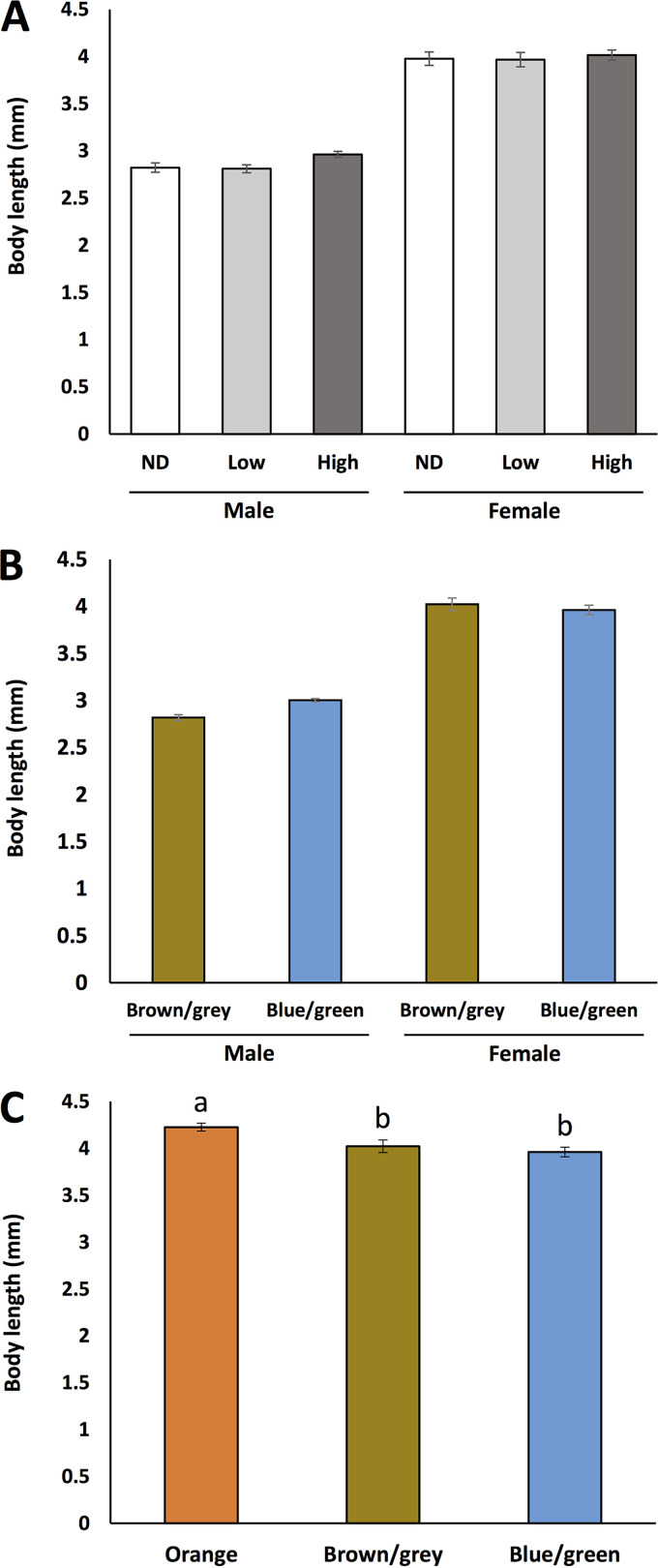
(A and B) Associations between body lengths of male and female *Macrohomotoma gladiata* with (A) different Liberibacter infection statuses and (B) different abdominal colors (for the females, only blue/green and brown/grey morphotypes were included in the initial statistical analysis). A three-way ANOVA (gender × abdominal color × infection status) showed that only gender was a significant factor associated with body length. (C) When including the orange morphotype and reexamining the association between abdominal color and body length in females, the results showed that body lengths of the orange morphotype were significantly larger than those of the others. Different letters indicate significant difference (Tukey’s HSD; *P < *0.05).

The associations among Liberibacter infection status and *M. gladiata* abdominal color and body length were evaluated by analysis of variance (ANOVA) tests (Fig. 5). When only analyzing the brown/grey and blue/green morphotypes in both genders (using a three-way ANOVA test), body length was not associated with Liberibacter infection density and abdominal color (Fig. 5 and Fig. S1). Gender was the only significant factor associated with body length (*P < *0.001); females were significantly larger than males.

When comparing the body lengths in all three morphotypes of females (using a two-way ANOVA test; Fig. 5C), there were significant differences among different morphotypes (*P = *0.026); the body lengths of the orange morphotype were significantly larger than that of the other morphotypes (Tukey’s honestly significant difference [HSD] test; *P < *0.05).

### Liberibacter infection patterns in *F*. *microcarpa* are associated with the presence of *M. gladiata* nymphs.

**FIG 6:**
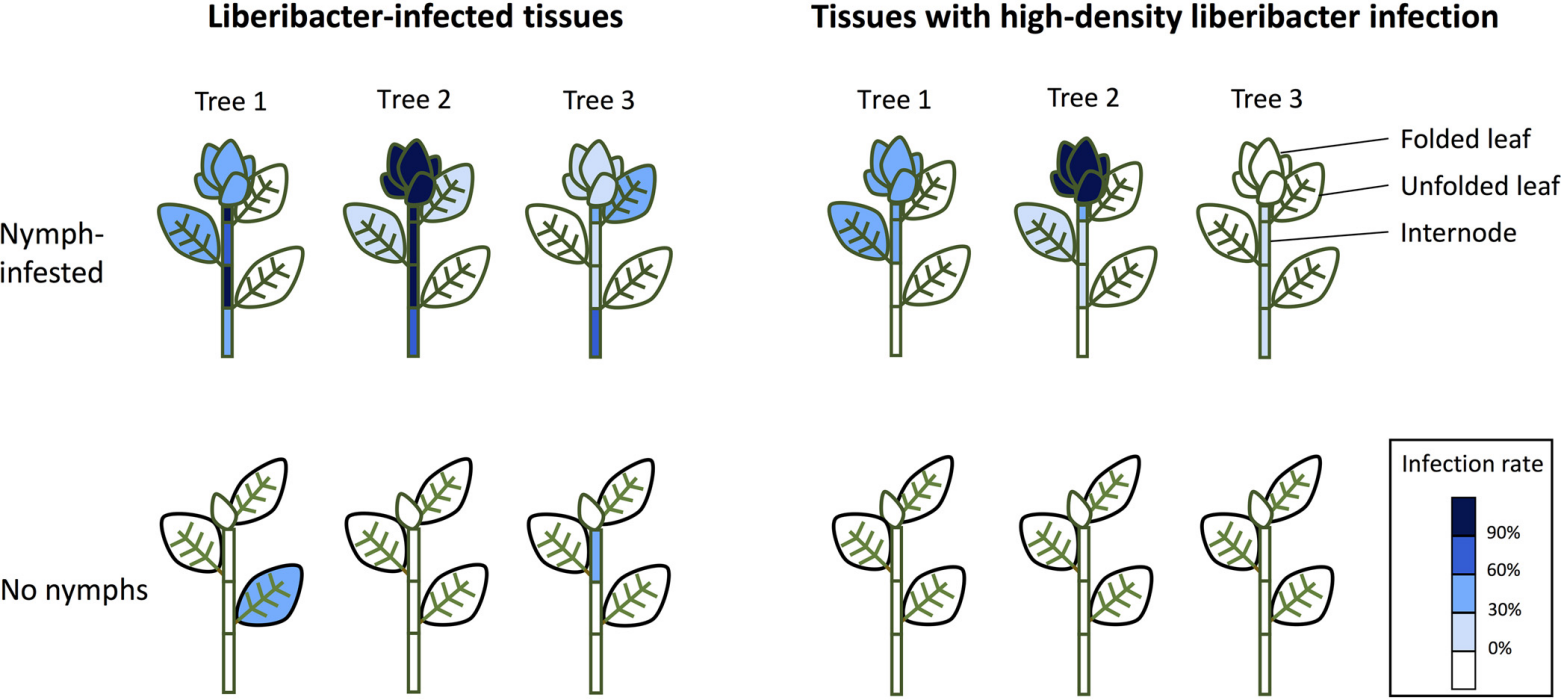
Liberibacter infection patterns in *Ficus microcarpa* tissues collected from three trees. At least three nymph-infested and three uninfested twigs were collected from each tree. Each twig was portioned into various parts for testing: folded leaves, the three topmost unfolded leaves, and the internodes between these leaves. The color coding indicates the proportion of samples that were Liberibacter positive (left) or carried high Liberibacter loads (right) in each sample type (in a specific tree).

To investigate the Liberibacter infection patterns in *F*. *microcarpa*, twig samples were collected from three trees (tree 1, tree 2, and tree 3) located in the West District, Taichung City. The trees appeared vigorous and did not show symptoms resembling those of known Liberibacter-associated diseases (4, 5). From each tree, at least three nymph-infested and three uninfested twigs were collected. All sampled twigs were partitioned into parts: the folded leaves at the tips of the twigs, the first, second, and third unfolded leaves (counting from the tip), and the internodes between each sampled leaf. The DNA samples obtained from different plant tissues were subjected to qPCR testing, and for each sample, the Liberibacter gene’s copy number was divided by that of the plant’s *actin 1* gene, and the resulting infection density value was grouped into one of the three categories described above. Liberibacter was detected in both infested and uninfested twigs (Fig. 6). Out of 11 nymph-infested twigs, 9 were Liberibacter positive (tree 1, 3/3; tree 2, 4/4; tree 3, 2/4); 2 out of 9 uninfested twigs were also Liberibacter positive (tree 1, 1/3; tree 2, 0/3; tree 3, 1/3). The percentages of samples that were Liberibacter positive varied among twigs and tissue types. However, in general, tissues collected from twigs infested with *M*. *gladiata* nymphs tend to have higher chances of being Liberibacter-positive (Fig. 6). The tissue types with the highest infection frequencies were either the nymph-infested folded leaves, the internodes, or the unfolded leaves (Fig. 6). None of the shoot tips in the uninfested twigs were Liberbacter-positive. In all three trees, high-density infection by Liberibacter was only detected in nymph-infested twigs (Fig. 6). The tissue types with the highest infection rates varied among infested twigs; they were either the infested, folded leaves, the internodes, or the unfolded leaves (Fig. 6). Additional PCR testing (with LG774F/LG1463R) and Sanger sequencing revealed that the sequences obtained from the three trees tested shared 100% identity (611/611 bp) with the Liberibacter detected in *M. gladiata*.

To confirm that *M*. *gladiata* nymphs attached to the infested twigs could carry Liberibacter, nymphs were collected from infested twigs and their DNA was tested with conventional PCR using LG774F/LG1463R. Five out of nine nymph DNA samples tested positive for Liberibacter infection (3, 2, and 0 Liberibacter-positive samples for tree 1 (*n* = 6), tree 2 (*n* = 2), and tree 3 (*n* = 1), respectively).

## DISCUSSION

In a recent study, researchers detected the presence of a Liberibacter in a few *M. gladiata* samples using Illumina sequencing; phylogenetic analyses based on 16S rDNA fragments of around 402 bp suggested that the bacterium was closely associated with *Ca*. Liberibacter asiaticus (31). In this work, phylogenetic analysis based on near-full-length 16S rDNA sequences resulted in the same phylogenetic placement of the Liberibacter (Fig. 2). Despite the consistency between the phylogenetic analysis results from the two studies, alignment of our 1,421-bp sequence against the 402-bp fragment from the previous study (31) revealed that there were three base differences (Fig. S2). Such differences may be due to intragenomic heterogeneity of different 16S rDNA copies or to sequencing errors produced during Illumina sequencing. Nevertheless, considering that 16S rDNA sequences (obtained by PCR using genus-specific primers) from all 40 individuals tested in this study were identical, it is evident that there is one dominant Liberibacter infecting *M. gladiata* in Taiwan, which is widespread across different populations. Over the past years *F*. *microcarpa* has been imported to many countries, and *M. gladiata* could potentially become an invasive species in these regions (27); the present work’s finding that a Liberibacter could be found in all of the tested *M*. *gladiata* populations suggests that the same Liberibacter could also be found outside of Taiwan.

The Liberibacter infection patterns from the qPCR assays varied significantly across populations (Fig. 3). Interpopulation variability in *Ca*. Liberibacter asiaticus has also been reported in *D*. *citri*. For *Ca*. Liberibacter asiaticus, bacterial titers can continue to increase in nymphs postacquisition; the extent of *Ca*. Liberibacter asiaticus proliferation also depends on whether acquisition occurred at the nymphal stage or not (34–36). Since only field-collected adults were directly tested in this study, it is possible that differences in psyllid age as well as when they acquired and how long they harbored the Liberibacter may contribute to the patterns observed in this work. Also, the plants that the psyllids inhabit may also carry different Liberibacter loads, subsequently affecting Liberibacter titers and infection frequency in each population. Finally, Liberibacter loads in different parts of the same tree may also vary; a previous study on *Ca*. Liberibacter asiaticus showed that petioles and midribs tend to carry higher Liberibacter titers than other plant parts (37). It is possible that Liberibacter titers in *M*. *gladiata* may also be influenced by such factors.

During the identification of potential factors affecting Liberibacter infection density, none of the collected male psyllids had orange abdomens. In *D*. *citri*, orange abdomens were generally only found in older males, usually 40 days after eclosion under laboratory conditions (38). The scarcity of male psyllids living past this time span (39) may explain why orange-morphotype males were not found in the field sampling conducted in this work.

Compared to females, male *M*. *gladiata* had higher proportions of individuals infected with high densities of Liberibacter (Fig. 4). This pattern is different from past studies on *D*. *citri*, which showed that the acquisition and titers of *Ca*. Liberibacter asiaticus were not associated with psyllid gender (40, 41). The abdominal color of *M*. *gladiata* was also found to be associated with Liberibacter infection density. Among the three morphotypes, psyllids with a blue/green abdomen had the highest infection density (Fig. 4B). Such a pattern is inconsistent with that of a previous work, in which *D*. *citri* individuals with a blue/green abdomen had lower Liberibacter titers (42). The blue/green abdominal color in *D*. *citri* has been attributed to the production of hemocyanin, an immune protein (43), and it was hypothesized that higher levels of hemocyanin in the blue/green morphotype could lead to lower Liberibacter loads (43). The findings in this work indicate that the association between psyllid-related factors and Liberibacter infection could vary depending on psyllid-Liberibacter combinations and highlight the importance of exploring similar interactions in other psyllid species.

Similar to a previous study (44), the data in this work showed that females of *M. gladiata* had larger body lengths than males. While no differences in body lengths were detected between blue/green and brown/grey morphotypes in both genders, the data showed that in females, individuals with a orange abdomen had significantly larger body lengths than the other morphotypes (Fig. 5C). In *D. citri*, orange abdomens are often observed in gravid females, and the eggs they carry may result in greater weight than males (45). However, virgin female psyllids have also been found with orange abdomens, suggesting that the eggs may not always be associated with the orange coloration (38) and perhaps body mass. Whether the orange abdominal color in *M*. *gladiata* females reflects their sexual maturity deserves further research.

Past reports indicated that some Liberibacters could alter their psyllid hosts’ fitness. It was found that *D. citri* infected by *Ca*. Liberibacter asiaticus exhibit higher fertility than their uninfected counterparts; however, the psyllids’ life spans were also reduced as a result of Liberibacter infection (14). In *B*. *cockerelli*, infection by *Ca*. Liberibacter solanacearum tends to reduce the insect hosts’ fecundity (14, 17). Since body length/size is often considered a determinant of insect fitness (46), the present study measured *M*. *gladiata* body length to examine whether Liberibacter infection status is associated with *M*. *gladiata* fitness. Based on the findings in this study (Fig. 5A), there is no evidence suggesting that the Liberibacter in *M. gladiata* could affect the insect’s fitness. Additional testing of other parameters indicative of *M. gladiata* fecundity in infected and uninfected individuals is needed to confirm whether Liberibacter could have an effect on other biological aspects of *M*. *gladiata*.

The distribution of Liberibacter in *F*. *microcarpa* was highly uneven and variable. However, high infection density was only detected in twigs whose shoot tips were infested with *M*. *gladiata* nymphs (Fig. 6). This suggests that the distribution of Liberibacter is associated with psyllid feeding. Similar patterns have also been found in other Liberibacter-associated disease systems. For example, the presence of *Ca.* Liberibacter americanus is more frequently detected in the distal ends of citrus trees than in the proximal ends (47); it has been suggested that flush tissues in the distal ends are more tender and appealing to psyllids during feeding, and psyllids tend to exhibit more active and longer probing behavior on new flushes than on mature leaves (48).

In terms of Liberibacter transmission among psyllids, a recent study on *D*. *citri* showed that the Liberibacter deposited by ovipositing females onto the flush during feeding could be a main source of Liberibacter transmission for the nymphs (49). The data in this work showed that twigs that carry the nymphs (thus including the ovipositional sites) tend to harbor higher titers of Liberibacter (Fig. 6) and that the Liberibacter could also be detected in nymphs. Therefore, it is likely that the mating and ovipositing adults or the nymphs are the main source of Liberibacter in *F*. *microcarpa* tissues, and that the Liberibacter in *M*. *gladiata* and *Ca*. Liberibacter asiaticus rely on similar strategies to spread among insect hosts. The consistency between our data and those of other studies (49) indicate that interfering with such a route of transmission could be an effective approach for reducing Liberibacter inoculum in the field.

Although Liberibacter could be consistently detected in *F*. *microcarpa*, the trees from which the samples were collected all exhibited vigorous growth, suggesting that the Liberibacter detected is not pathogenic to the plant. Indeed, previous studies have shown that some Liberibacter are not associated with plant diseases (6, 7). For example, high titers of “*Ca.* Liberibacter europaeus” have been detected in pear trees without there being symptoms (6); such a bacterium was considered an endophyte of the plant (6), and this could also be the case for the Liberibacter detected in *F*. *microcarpa*.

Overall, the present study shed light on the infection patterns of a Liberibacter associated with *M*. *gladiata* and *F*. *microcarpa*. The findings showed that the Liberibacter could exhibit host-microbe associations similar to or distinct from those of other well-known Liberibacters. Understanding infection patterns that are consistent among different Liberibacter-psyllid systems could help broaden our understanding of this interesting group of bacteria and help devise strategies for managing plant diseases associated with them.

## MATERIALS AND METHODS

### Sampling of psyllids and plant tissues.

All adult psyllids used in this work were hand-caught in the field. The sampling sites were all located in urban areas. To study the Liberibacter infection patterns across different *M*. *gladiata* populations, psyllids were sampled in 2019 and 2021. A total of 58 *M*. *gladiata* adults were collected from three locations in Taichung, Taiwan (TC1, TC2, and TC3) in 2019; a total of 25 and 12 adults were then collected in Tainan (TN) and Taipei (TP), respectively, in 2021. All insect samples were recorded for their gender and stored at −80°C prior to further experiments.

In 2020, a larger set of samples (*n* = 238) was collected for identifying potential factors associated with Liberibacter infection status. For this sample set, the gender, abdominal color, and body length (head to abdomen) values were recorded for each insect prior to storage at −80°C. The abdominal colors of the psyllids were grouped into three categories: orange, brown/grey, and blue/green.

To investigate the Liberibacter infection patterns in *F*. *microcarpa*, twigs including at least three unfolded leaves were collected from three trees (tree 1, tree 2, and tree 3) located in the West District, Taichung City (24°08′09.7″N 120°40′30.2″E) in 2021. Three, four, and four nymph-infested twigs were collected from tree 1, tree 2, and tree 3, respectively. Three uninfested twigs were also collected from each tree.

Additionally, nymphs attached to the infested twigs were also sampled, and their DNA samples were obtained using the methods mentioned above. Only live, larger nymphs were collected. Six, two, and one nymph were collected from tree 1, tree 2, and tree 3, respectively.

### DNA extraction.

Psyllid DNA was extracted using the DNeasy blood and tissue kit (Qiagen, Inc., Hilden, Germany); each sample was extracted from a single psyllid. All DNA samples were examined for their qualities and concentrations using the Nanodrop One spectrophotometer (Thermo Fisher Scientific, Waltham, MA) and then diluted to 30 ng/μL or 20 ng/μL, depending on the lowest concentration detected in each sample set. DNA samples used for surveying the Liberibacter infection patterns in different *M. gladiata* populations were diluted to 30 ng/μL; for assays identifying potential factors associated with Liberibacter infection densities, the samples were diluted to 20 ng/μL. Samples extracted from nymphs were diluted to 30 ng/μL.

Plant DNA was extracted from 0.01 to 0.05 g of plant tissue using the Synergy plant DNA extraction kit (OPS Diagnostics, Lebanon, NJ). The leaf samples included midrib and lamina tissues. All samples were diluted to 10 ng/μL.

### Detection of Liberibacter using genus-specific primers.

Conventional PCR tests using Liberibacter-specific primers (7) LG774F (5′-GTAAACGATGAGTGCTAGCTGTTGGG-3′) and LG1463R (5′-CTGACCRTACCGTGGCCGG-3′) were conducted on psyllid DNA samples obtained from different populations (Table 1). The expected amplicon was about 684 bp in size. The thermal-cycling conditions were set as previously described (7). All conventional PCR assays in this study were completed using GoTaq green master mix (Promega, Madison, WI). Each 25-μL PCR included 0.25 μM each primer and 30 ng of template DNA.

After examination of the PCR results using gel electrophoresis, the proportion of Liberibacter-positive samples at each sampling site was recorded, and the Liberibacter-specific amplicons produced were recovered using a Zymoclean gel DNA recovery kit (Zymo Research, Irvin, CA) and subjected to Sanger sequencing. The obtained sequences were imported into Geneious R10 (Biomatters Ltd., Auckland, New Zealand) and compared against each other. Thereafter, the sequences were searched against the National Center for Biotechnology Information (NCBI) GenBank database using BLASTn (50).

### Cloning and analysis of Liberibacter near-full-length 16S rDNA sequence.

The thermal cycling conditions for PCR tests using bacterial universal 16S rRNA primers 27f (5′-AGAGTTTGATCMTGGCTCAG-3′) and 1492r (5′-GGYTACCTTGTTACGACTT-3′) (33) were 95°C for 3 min, followed by 35 cycles of 95°C for 30 s, 55°C for 30 s, 72°C for 1 min 30 s, and a final extension at 72°C for 5 min. The volume for these reactions was 25 μL; each reaction included 0.4 μM each primer and 30 ng of DNA. The amplicons were cloned using the pgem-t easy vector system (Promega, Madison, WI). Clones were screened using LG774F/LG1463R. Four and three clones carrying a Liberibacter 16S rDNA fragment were sequenced for the two psyllid DNA samples, respectively. The obtained consensus sequence (1,421 bp) was aligned with those of other known Liberibacters using CLUSTAL W (51), and the alignment was used to reconstruct a maximum-likelihood tree in MEGA11 (52). The Liberibacter sequences included in this analysis (Table S2) were those mentioned in previous work (7, 31); only sequences with sufficient lengths and able to align with the sequence from this work were included. The analysis was conducted using the K2+G+I model with 1,000 replicates of bootstrap analysis.

### Quantification of Liberibacter infection densities in psyllids.

Primers (HLBas: 5′-TCGAGCGCGTATGCGAATAC-3′; HLBr: 5′-GCGTTATCCCGTAGAAAAAGGTAG-3′) and probe (HLBp: 5′-AGACGGGTGAGTAACGCG-3′) developed in a previous study (53) were used to quantify the Liberibacter 16S rDNA copy numbers. Analysis of the 16S rDNA sequence in *M*. *gladiata* (described in detail below) showed that such an assay was applicable for the Liberibacter identified in this work. To normalize the Liberibacter copy numbers, primers GKF (5′-CTGGAGTGGGACCCAATGCT-3′) and GKR (5′-GCAGCTTGCTGATTGCCCAA-3′), which were specific to *M. gladiata*’s glycerol kinase gene, were designed based on a transcriptomic data set (GenBank accession number GCXQ01037858); the copy number of *M. gladiata*’s glycerol kinase gene in each sample was determined using SYBR green assays.

To determine the gene copy numbers in the DNA samples, plasmid-based standard curves were constructed during each qPCR run. Plasmids carrying different target gene fragments were obtained prior to the qPCR assays. For the Liberibacter assays, a plasmid carrying the near-full-length 16S rDNA fragment of the target Liberibacter was selected from the clone library (described above) and used throughout the experiments. For the psyllid gene, the target fragment was amplified using GKF and GKR and cloned into the pGEM-T Easy Vector. The thermal cycling conditions for the assay were 95°C for 5 min, followed by 30 cycles of 95°C for 30 s, 60°C for 30 s, 72°C for 1 min, and a final extension at 72°C for 5 min. The reaction was 25 μL in volume and included 0.4 μM each primer and 30 ng of DNA. Prior to qPCR testing, plasmid DNA was extracted from an overnight culture of an Escherichia coli transformant using the QIAprep spin miniprep kit (Qiagen, Inc., Hilden, Germany). Plasmid DNA was then digested using PstI-HF (New England Biolabs, Ipswich, MA) and purified using the QIAquick gel extraction kit (Qiagen, Inc.). The recovered plasmid DNA was quantified with a Nanodrop One spectrophotometer and stored until use. During each qPCR run, a linearized plasmid sample was serially diluted, and the diluted samples were tested along with the DNA samples. To ensure that data across different assays were comparable with each other, we made sure that the plasmids used for each gene in each set of experiments originated from the same preparation of linearized plasmids.

All qPCR assays in this work were conducted using a CFX-Connect real-time PCR system (Bio-Rad Laboratories, Inc., Hercules, CA). For TaqMan assays, the iTaq universal probe supermix (Bio-Rad Laboratories, Inc.) was used. All SYBR green assays were conducted using the iQ SYBR green supermix (Bio-Rad Laboratories, Inc.). Each plasmid or genomic DNA sample included three technical replicates. The reaction volumes for all the assays were 25 μL. Each TaqMan reaction included 0.1 μM each primer and 0.1 μM probe; each SYBR green reaction contained 0.4 μM each primer. In assays evaluating Liberibacter infection patterns in different *M. gladiata* populations, each reaction contained 30 ng of DNA. For assays identifying potential psyllid-associated factors affecting Liberibacter infection density, 20 ng of DNA was added to each reaction.

The cycling conditions for the TaqMan assays were 95°C for 3 min followed by 40 cycles of 95°C for 10 s and 60°C for 30 s. The cycling conditions for the SYBR green assays were 95°C for 3 min followed by 40 cycles of 95°C for 10 s and 58°C for 30 s. A dissociation curve analysis was conducted at the end of each SYBR green assay. Data collected from assays with acceptable PCR efficiencies (81.1 to 93.8%; *R*^2^ > 0.98) were subjected to further analyses.

### Investigation of Liberibacter infection patterns in *F*. *microcarpa*.

Quantification of Liberibacter using TaqMan assays was conducted as described above. The amount of template DNA used for each reaction was 10 ng. To normalize the data, primers AGF (CATGCCATCCTCCGTCTTGA) and AGR (ACTGAGGAACTGCTCTTGGC), which are specific to *Ficus microcarpa*’s *actin 1* gene (GenBank accession number JQ820243) were designed.

SYBR green assays using primer pair AGF/AGR were conducted to quantify the *actin 1* gene’s copy numbers. The reaction volume for this assay was 25 μL. Each reaction contained 0.4 μM each primer and 10 ng of DNA. The amplification conditions were 95°C for 3 min, followed by 40 cycles of 95°C for 10 s and 60°C for 30 s. A melt curve analysis was then conducted.

To ensure that the Liberibacter detected in the trees was the same as that in *M*. *gladiata*, three Liberibacter-positive samples (each extracted from unfolded leaves of a psyllid-infested twig from a different tree) were subjected to amplification by primer pair LG774F/LG1463R and sequenced. Each PCR included 10 ng of plant DNA. The assays were conducted as described above. The sequences detected in the trees were aligned and compared with those obtained from psyllid samples.

To confirm that *M*. *gladiata* nymphs collected from the trees could carry Liberibacter, the DNA samples of nymphs collected from infested twigs were also tested with LG774F/LG1463R using the methods described above. The amount of template DNA used for each reaction was 30 ng.

### Data analyses.

The copy numbers of each gene in each qPCR run were calculated based on previously described methods (54). After obtaining the infection density values and grouping the samples based on the Liberibacter infection categories, proportions of individuals belonging to different categories were compared across psyllid populations, genders, and abdominal colors using either the Fisher-Freeman-Halton test, Fisher’s exact test, or the Chi-square test, depending on the characteristics of the data. All statistical analyses were performed in SPSS (Bio-Rad Laboratories, Inc., Hercules, CA). Additional assessments of the associations between Liberibacter infection status, psyllid abdominal color, and body length were conducted using two-way or three-way analysis of variance (ANOVA) tests.

### Data availability.

The 16S rDNA gene sequences of the Liberibacter detected in *M*. *gladiata* have been deposited in GenBank under accession numbers OP159066 and OP159054.
