# Supplementary material for: Infection Patterns of a Liberibacter Associated with Macrohomotoma gladiata, a Psyllid Feeding on Ficus microcarpa
Source: Microbiol Spectr. 2022 Dec 1;10(6):e03614-22. doi: 10.1128/spectrum.03614-22 (PMC9769916; doi:10.1128/spectrum.03614-22)
Supplement: Supplemental file 1 — Fig. S1 and S2 and Tables S1 and S2. Download spectrum.03614-22-s0001.pdf, PDF file, 0.5 MB [file spectrum.03614-22-s0001.pdf]

SUPPLEMENTAL MATERIAL

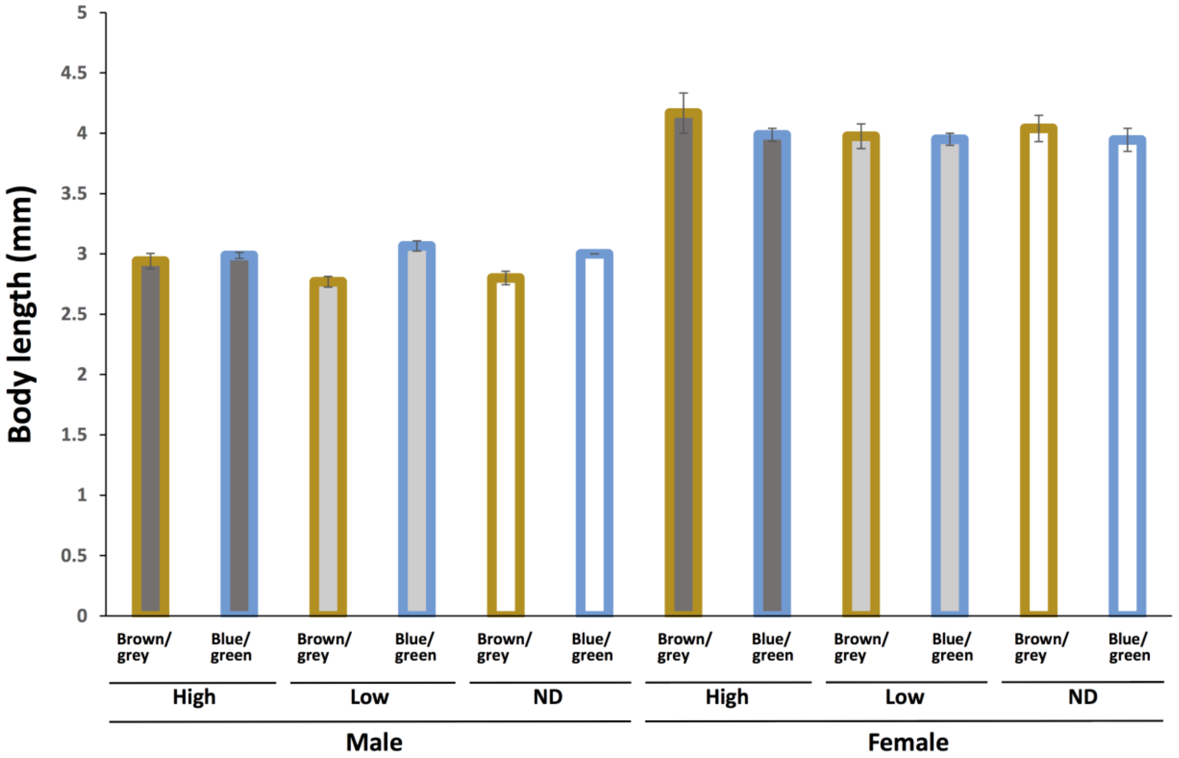

Fig. S1. Body lengths of male and female *Macrohomotoma gladiata* with different abdominal colors (brown/grey or blue/green) and Liberibacter infection statuses [(high, low or ND (undetected)]. A three-way ANOVA (gender x abdominal color x infection status) showed that only gender was a significant factor associated with body length.

```
OP159054.1      1 TGGGGAATATTGGACAATGGGGGCAACCCTGATCCAGCCATGCCGCGTGAGTGAAGAAGG  60
                  TGGGGAATATTGGACAATGGGGGCAACCCTGATCCAGCCATGCCGCGTGAGTGAAGAAGG
OL221597.1      1 TGGGGAATATTGGACAATGGGGGCAACCCTGATCCAGCCATGCCGCGTGAGTGAAGAAGG  60

OP159054.1     61 CCTTAGGGTTGTAAAGCTCTTTCGCCGGAGAAGATAATGACGGTATTCGGAGAAGAAGCC 120
                  CCTTAGGGTTGTAAAGCTCTTTCGCCGGAGAAGATAATGACGGTATTCGGAGAAGAAGCC
OL221597.1     61 CCTTAGGGTTGTAAAGCTCTTTCGCCGGAGAAGATAATGACGGTATTCGGAGAAGAAGCC 120

OP159054.1    121 CCGGCTAACTTCGTGCCAGCAGCCGCGGTAATACGAAGGGGGCGAGCGTTGTTCGGAATA 180
                  CCGGCTAACTTCGTGCCAGCAGCCGCGGTAATACGAAGGGGGCGAGCGTTGTTCGGAATA
OL221597.1    121 CCGGCTAACTTCGTGCCAGCAGCCGCGGTAATACGAAGGGGGCGAGCGTTGTTCGGAATA 180

OP159054.1    181 ACTGGGCGTAAAGGGCGCGTAGGCGGGCGATTAAGTTAAGGGTGAAATCCCAGGGCTTAA 240
                  ACTGGGCGTAAAGGGCGCGTAGGCGGGCGATTAAGTTAAGGGTGAAATCCCAGGGCTTAA
OL221597.1    181 ACTGGGCGTAAAGGGCGCGTAGGCGGGCGATTAAGTTAAGGGTGAAATCCCAGGGCTTAA 240

OP159054.1    241 CCCTGGAACTGCCTTTAATACTGGTTGTCTAGAGTTCAGGAGAGGTGAGTGGAATTCCGA 300
                  CCCTGGAACTGCCTTTAATACTGGTTGTCTAGAGTTCAGGAGAGGTGAGTGGAATTCCGA
OL221597.1    241 CCCTGGAACTGCCTTTAATACTGGTTGTCTAGAGTTCAGGAGAGGTGAGTGGAATTCCGA 300

OP159054.1    301 GTGTAGAGGTGAAATTCGTAGATATTCGGAGGAACACCGGTGGCGAAGGCGGCTCACTGG 360
                  GTGTAGAGGTGAAATTCGTAGATATTCGGAGGAACACCGGTGGCGAAGGCGGCTCACTGG
OL221597.1    301 GTGTAGAGGTGAAATTCGTAGATATTCGGAGGAACACCGGTGGCGAAGGCGGCTCACTGG 360
                                 ⬇ ⬇ ⬇
OP159054.1    361 CCTGATACTGACGCTGAGGCGCGAAAGCGTGGGGAGCAAACA 402
                  CCTGATACTGACGCT A G GCGAAAGCGTGGGGAGCAAACA
OL221597.1    361 CCTGATACTGACGCTCATGTGCGAAAGCGTGGGGAGCAAACA 402
```

Fig. S2. Alignment between the Liberibacter sequence detected in this study (accession no. OP159054.1) and that detected in a previous work by Kwak et al. (2021; accession no. OL221597.1). The base differences are indicated with dark arrows.

Table S1. Sequences used in the phylogenetic analysis

Species (strain/isolate)	Accession no.
Agrobacterium fabacearum strain Ach5	NZ_CP011246.1
Candidatus Liberibacter africanus strain PTSAPSY	CP004021.1
Candidatus Liberibacter africanus strain Mpumalanga-UPCRI-06-0071	EU754741.1
Candidatus Liberibacter americanus strain Sao Paulo	CP006604.1
Candidatus Liberibacter asiaticus strain gxpsy	CP004005.1
Candidatus Liberibacter asiaticus strain Ishi-1	AP014595.1
Candidatus Liberibacter asiaticus strain psy62	CP001677.5
Candidatus Liberibacter asiaticus strain A4	CP010804.2
Candidatus Liberibacter brunswickensis isolate Asol15	KY077741.1
Candidatus Liberibacter europaeus isolate 94B	JX244260.1
Candidatus Liberibacter europaeus isolate ASNZ1 CLeu_NZ1_02	PSQJ01000002.1
Candidatus Liberibacter europaeus isolate BrS	JX244259.1
Candidatus Liberibacter europaeus isolate Psy6	JX244258.1
Candidatus Liberibacter psyllaurous isolate PRR1	EU812559.1
Candidatus Liberibacter solanacearum strain CLso-ZC1	CP002371.1
Candidatus Liberibacter solanacearum isolate NZ082226	EU834130.1
Candidatus Liberibacter solanacearum isolate NZ083338	EU935004.1
Candidatus Liberibacter solanacearum strain LsoNZ1 CLso_NZ1	JMTK01000002.1
Candidatus Liberibacter africanus 16S strain LEI16SRRNB	L22533.1
Liberibacter crescens strain BT-1	CP003789.1
Candidatus Liberibacter ctenarytainae isolate Oxford CLct_Ox_contig17	SEOL01000017.1

Table S2. GenBank entries sharing high sequence similarities with the liberibacter 16S rDNA (611 bp) detected in this work

Name of species and strain	Percent identity	Accession no.
Uncultured *Candidatus* Liberibacter sp. clone DS318	98.2	FJ388691.1
Uncultured *Candidatus* Liberibacter sp. clone DS162	98.2	FJ388535.1
Uncultured *Candidatus* Liberibacter sp. clone DS75	98.2	FJ388448.1
Candidatus Liberibacter asiaticus strain A4	98.04	CP010804.2
Candidatus Liberibacter asiaticus strain JXGC	98.04	CP019958.1
Candidatus Liberibacter asiaticus strain ReuSP1	98.04	CP061535.1
Candidatus Liberibacter asiaticus str. Ishi-1	98.04	AP014595.1
Candidatus Liberibacter asiaticus strain GDHZ11D	98.04	CP045565.1
Uncultured *Candidatus* Liberibacter sp. clone Cl-DOTU10	98.04	KJ019524.1
Uncultured *Candidatus* Liberibacter sp. clone Cl-DOTU09	98.04	KJ019523.1
Candidatus Liberibacter asiaticus strain Myan16	98.04	CP060689.1
Candidatus Liberibacter asiaticus str. gxpsy	98.04	CP004005.1
Uncultured bacterium clone OTU25	98.04	JN224954.1
Uncultured bacterium clone OTU36	98.04	JN224952.1
Uncultured bacterium clone OTU28	98.04	JN224944.1
Uncultured bacterium clone OTU26	98.04	JN224942.1
Candidatus Liberibacter asiaticus isolate TaiYZ2	98.04	CP041385.1
Candidatus Liberibacter asiaticus isolate CoFLP	98.04	CP054558.1
Candidatus Liberibacter asiaticus isolate JRPAMB1	98.04	CP040636.1
Candidatus Liberibacter asiaticus str. psy62	98.04	CP001677.5
Uncultured bacterium clone T2S-T7-A03	98.04	GU166675.1
Candidatus Liberibacter asiaticus isolate OK901	98.04	AB480072.1
Candidatus Liberibacter asiaticus clone H04	98.04	GQ254633.1
Candidatus Liberibacter asiaticus clone H03	98.04	GQ254632.1
Candidatus Liberibacter asiaticus clone H02	98.04	GQ254631.1
Candidatus Liberibacter asiaticus clone H01	98.04	GQ254630.1
Candidatus Liberibacter asiaticus clone G04	98.04	GQ254629.1
Candidatus Liberibacter asiaticus clone G02	98.04	GQ254627.1
Candidatus Liberibacter asiaticus clone G01	98.04	GQ254626.1
Candidatus Liberibacter asiaticus clone F03	98.04	GQ254624.1
Candidatus Liberibacter asiaticus clone F01	98.04	GQ254622.1
Candidatus Liberibacter asiaticus clone E01	98.04	GQ254618.1
Candidatus Liberibacter asiaticus clone B03	98.04	GQ254609.1
Candidatus Liberibacter asiaticus clone B01	98.04	GQ254608.1
Candidatus Liberibacter asiaticus clone A03	98.04	GQ254606.1
Candidatus Liberibacter asiaticus clone A02	98.04	GQ254605.1
Candidatus Liberibacter asiaticus clone A01	98.04	GQ254604.1
Uncultured *Candidatus* Liberibacter sp. clone DS501	98.04	FJ388874.1
Uncultured *Candidatus* Liberibacter sp. clone DS494	98.04	FJ388867.1
Uncultured *Candidatus* Liberibacter sp. clone DS492	98.04	FJ388865.1
Uncultured *Candidatus* Liberibacter sp. clone DS488	98.04	FJ388861.1
Uncultured *Candidatus* Liberibacter sp. clone DS479	98.04	FJ388852.1

Table S2 (continued)

Uncultured *Candidatus* Liberibacter sp. clone DS472	98.04	FJ388845.1
Uncultured *Candidatus* Liberibacter sp. clone DS466	98.04	FJ388839.1
Uncultured *Candidatus* Liberibacter sp. clone DS458	98.04	FJ388831.1
Uncultured *Candidatus* Liberibacter sp. clone DS457	98.04	FJ388830.1
Uncultured *Candidatus* Liberibacter sp. clone DS451	98.04	FJ388824.1
Uncultured *Candidatus* Liberibacter sp. clone DS448	98.04	FJ388821.1
Uncultured *Candidatus* Liberibacter sp. clone DS446	98.04	FJ388819.1
Uncultured *Candidatus* Liberibacter sp. clone DS412	98.04	FJ388785.1
Uncultured *Candidatus* Liberibacter sp. clone DS403	98.04	FJ388776.1
Uncultured *Candidatus* Liberibacter sp. clone DS401	98.04	FJ388774.1
Uncultured *Candidatus* Liberibacter sp. clone DS390	98.04	FJ388763.1
Uncultured *Candidatus* Liberibacter sp. clone DS386	98.04	FJ388759.1
Uncultured *Candidatus* Liberibacter sp. clone DS338	98.04	FJ388711.1
Uncultured *Candidatus* Liberibacter sp. clone DS332	98.04	FJ388705.1
Uncultured *Candidatus* Liberibacter sp. clone DS328	98.04	FJ388701.1
Uncultured *Candidatus* Liberibacter sp. clone DS312	98.04	FJ388685.1
Uncultured *Candidatus* Liberibacter sp. clone DS309	98.04	FJ388682.1
Uncultured *Candidatus* Liberibacter sp. clone DS307	98.04	FJ388680.1
Uncultured *Candidatus* Liberibacter sp. clone DS303	98.04	FJ388676.1
Uncultured *Candidatus* Liberibacter sp. clone DS292	98.04	FJ388665.1
Uncultured *Candidatus* Liberibacter sp. clone DS270	98.04	FJ388643.1
Uncultured *Candidatus* Liberibacter sp. clone DS253	98.04	FJ388626.1
Uncultured *Candidatus* Liberibacter sp. clone DS252	98.04	FJ388625.1
Uncultured *Candidatus* Liberibacter sp. clone DS240	98.04	FJ388613.1
Uncultured *Candidatus* Liberibacter sp. clone DS235	98.04	FJ388608.1
Uncultured *Candidatus* Liberibacter sp. clone DS223	98.04	FJ388596.1
Uncultured *Candidatus* Liberibacter sp. clone DS212	98.04	FJ388585.1
Uncultured *Candidatus* Liberibacter sp. clone DS208	98.04	FJ388581.1
Uncultured *Candidatus* Liberibacter sp. clone DS197	98.04	FJ388570.1
Uncultured *Candidatus* Liberibacter sp. clone DS193	98.04	FJ388566.1
Uncultured *Candidatus* Liberibacter sp. clone DS192	98.04	FJ388565.1
Uncultured *Candidatus* Liberibacter sp. clone DS191	98.04	FJ388564.1
Uncultured *Candidatus* Liberibacter sp. clone DS173	98.04	FJ388546.1
Uncultured *Candidatus* Liberibacter sp. clone DS164	98.04	FJ388537.1
Uncultured *Candidatus* Liberibacter sp. clone DS160	98.04	FJ388533.1
Uncultured *Candidatus* Liberibacter sp. clone DS159	98.04	FJ388532.1
Uncultured *Candidatus* Liberibacter sp. clone DS94	98.04	FJ388467.1
Uncultured *Candidatus* Liberibacter sp. clone DS90	98.04	FJ388463.1
Uncultured *Candidatus* Liberibacter sp. clone DS82	98.04	FJ388455.1
Uncultured *Candidatus* Liberibacter sp. clone DS80	98.04	FJ388453.1
Uncultured *Candidatus* Liberibacter sp. clone DS67	98.04	FJ388440.1
Uncultured *Candidatus* Liberibacter sp. clone DS59	98.04	FJ388432.1
Uncultured *Candidatus* Liberibacter sp. clone DS49	98.04	FJ388422.1
Uncultured *Candidatus* Liberibacter sp. clone DS44	98.04	FJ388417.1
Uncultured *Candidatus* Liberibacter sp. clone DS40	98.04	FJ388413.1
Uncultured *Candidatus* Liberibacter sp. clone DS31	98.04	FJ388404.1

Table S2 (continued)

Uncultured *Candidatus* Liberibacter sp. clone DS30	98.04	FJ388403.1
Uncultured *Candidatus* Liberibacter sp. clone DS26	98.04	FJ388399.1
Uncultured *Candidatus* Liberibacter sp. clone DS23	98.04	FJ388396.1
Uncultured *Candidatus* Liberibacter sp. clone DS16	98.04	FJ388389.1
Uncultured *Candidatus* Liberibacter sp. clone DA172	98.04	FJ388357.1
Uncultured *Candidatus* Liberibacter sp. clone GS409	98.04	FJ388184.1
Uncultured *Candidatus* Liberibacter sp. clone GS391	98.04	FJ388166.1
Uncultured *Candidatus* Liberibacter sp. clone GS388	98.04	FJ388163.1
Uncultured *Candidatus* Liberibacter sp. clone GS379	98.04	FJ388154.1
Uncultured *Candidatus* Liberibacter sp. clone GS366	98.04	FJ388141.1
Uncultured *Candidatus* Liberibacter sp. clone GS364	98.04	FJ388139.1
Uncultured *Candidatus* Liberibacter sp. clone GS356	98.04	FJ388131.1
